# Hydrogen evolution reaction measurements of dealloyed porous NiCu

**DOI:** 10.1186/1556-276X-8-528

**Published:** 2013-12-17

**Authors:** Kyla R Koboski, Evan F Nelsen, Jennifer R Hampton

**Affiliations:** 1 Department of Physics, Hope College, Holland, MI 49423, USA; 2 Department of Physics and Astronomy, University of North Carolina at Chapel Hill, Chapel Hill, NC 27599, USA

**Keywords:** Dealloying, Alloy, NiCu, Ni, Cu, Hydrogen evolution reaction

## Abstract

Porous metals are of interest for their high surface area and potential for enhanced catalytic behavior. Electrodeposited NiCu thin films with a range of compositions were electrochemically dealloyed to selectively remove the Cu component. The film structure, composition, and reactivity of these samples were characterized both before and after the dealloying step using scanning electron microscopy, energy-dispersive spectroscopy, and electrochemical measurements. The catalytic behavior of the dealloyed porous Ni samples towards the hydrogen evolution reaction was measured and compared to that of the as-deposited samples. The dealloyed samples were generally more reactive than their as-deposited counterparts at low overpotentials, making the dealloying procedure a promising area of exploration for improved hydrogen evolution catalysts.

## Background

Nanoporous metal structures are of significant interest for a wide variety of applications due to their low density, high surface area, enhanced optical properties, and improved catalytic behavior [1]. Electrochemical dealloying of a metallic alloy has been used to produce a number of different nanoporous metals, including nickel [2-4], gold [5-12], copper [8,13,14], silver [8,15], iron [16], platinum [17], and palladium [18].

In most cases, during dealloying, the less noble (more thermodynamically active) component is selectively oxidized from the alloy, while the remaining material may rearrange to form an interconnected network of pores [19,20]. However, Searson and coworkers recently showed that the more noble component of an alloy can be selectively removed if more thermodynamically active component is kinetically stabilized. In particular, the nickel component of a NiCu alloy was passivated in the electrolyte chosen for the dealloying procedure, allowing copper to be electrochemically removed [21]. This demonstration, which has also been shown in other electrolytes [22,23], opens up a wider range of alloy combinations that can be electrochemically dealloyed to produce nanoporous materials.

Searson and coworkers used the results of NiCu dealloying to identify an interesting core/shell structure in the originally deposited alloy [24]. This structure was subsequently confirmed by spatially resolved composition measurements [25], and the kinetics of the deposition process that facilitates its formation was studied [26]. By combining this core/shell structure with deposition into nanoporous templates and selective dealloying, the fabrication of nickel nanotubes is possible [24,25,27].

The magnetic behavior of these dealloyed NiCu samples have been characterized [21,24,28]. Modifications have also been made to the nanoporous structure for specific intended applications. For example, they have been used as templates for the deposition of oxide materials to fabricate pseudocapacitors with high specific capacitance [29-34], for the deposition of silicon to fabricate high-capacity current collectors for battery applications [35], and for the deposition of silver for surface-enhanced Raman spectroscopy applications [36]. Small amounts of metallic palladium have been deposited on nanoporous nickel substrates, and the resulting catalytic activity towards methanol and ethanol oxidation was characterized [37].

Here we characterize the catalytic activity of dealloyed NiCu samples towards the hydrogen evolution reaction (HER). Efficient and cost-effective production of hydrogen is an important area of research for renewable and environmentally friendly energy technology. Nickel and nickel alloys show the potential to be lower-cost options for electrocatalysis of hydrogen production compared to other precious metals such as platinum [38-43]. Porous Ni films showing enhanced activity towards the HER have been produced by leaching of Zn and Al from NiZn [2,44-47] and NiAl [48-52] alloys respectively. However, the HER reactivity of porous Ni films produced from selective removal of Cu from NiCu has not yet been explored.

In this work, NiCu thin films with varying compositions were electrodeposited, and the copper was selectively removed via electrochemical dealloying. The structure, composition, and reactivity of the samples were characterized both before and after the dealloying step using scanning electron microscopy (SEM), energy-dispersive spectroscopy (EDS), and electrochemical measurements.

## Methods

### Deposition and dealloying

The gold wafers on which the NiCu was deposited were cleaved from a silicon wafer plated with 1,000 Å of gold over a 50 Å titanium adhesion layer (Platypus Technologies, LLC, Madison, WI, USA). The electrochemical measurements were completed using a BAS Epsilon Electrochemical Workstation (Bioanalytical Systems, Inc., West Lafayette, IN, USA) and a custom-built Teflon cell [53] with a defined working electrode area of 0.032 cm^2^, a platinum wire (Alfa Aesar, Ward Hill, MA, USA) counter electrode, and an Ag/AgCl (3 M NaCl) reference electrode (Bioanalytical Systems, Inc., West Lafayette, IN, USA). All potentials are reported with respect to the Ag/AgCl reference electrode. The electrolyte solutions were made using water that had been purified through successive reverse osmosis, deionization, and UV purification stages. All chemicals were purchased from Sigma-Aldrich (St. Louis, MO, USA) and used as received. All experiments were carried out at room temperature.

The films were deposited from 0.5 M H_3_BO_3_ and 1 M Na_2_SO_4_ solutions with varying NiSO_4_ and CuSO_4_ concentrations (the sum of which was held constant at 0.11 M). The potential of the working electrode was stepped from open circuit to -1,200 mV until a total 50 mC of charge had been deposited. The dealloying step was performed in a 1 M Na_2_SO_4_ solution using linear sweep voltammetry (LSV). The potential was swept from 0mV to between 2,100 and 2,400mV at a scan rate of 5mV/s.

### Characterization

Characterization of the composition, structure, and reactivity of all the samples was performed before and after the dealloying step. Electrochemical capacitance measurements were carried out in a 1 M Na_2_SO_4_ solution using cyclic voltammetry (CV). The potential was cycled from -250 to 0 mV back to -250 mV at scan rates from 25 to 400 mV/s. The average current for the forward and reverse scans was graphed vs. the scan rate to extract the observed capacitance, a measure of the effective area of the sample.

Measurement of the HER was performed in 1 M NaOH. The sample was first pretreated by the application of a constant current of 50 *μ*A for 5 min. Then, the HER measurement was completed by sweeping the potential from -1,400 to -1,200 mV at a scan rate of 5 mV/s. The potential vs. Ag/AgCl was converted to overpotential based on the standard electrode potential of the HER and the pH of the electrolyte [54], and the current density was calculated with respect to the geometric area of the sample [53]. The current vs. overpotential data were fit to the Tafel equation to obtain the Tafel slope and exchange current density for the measured HER [55].

SEM and EDS measurements were carried out using a TM3000 Tabletop SEM (Hitachi, Tokyo, Japan) with a Quantax 70 EDS attachment (Bruker, Madison, WI, USA). Images were taken over a variety of field view sizes from ×60 to ×30,000 magnification. Composition measurements were extracted from EDS spectra taken at ×250 magnification, and Quantax 70 software was used to extract Ni and Cu compositions from the spectra.

## Results and discussion

### Deposition and dealloying results

**Figure 1:**
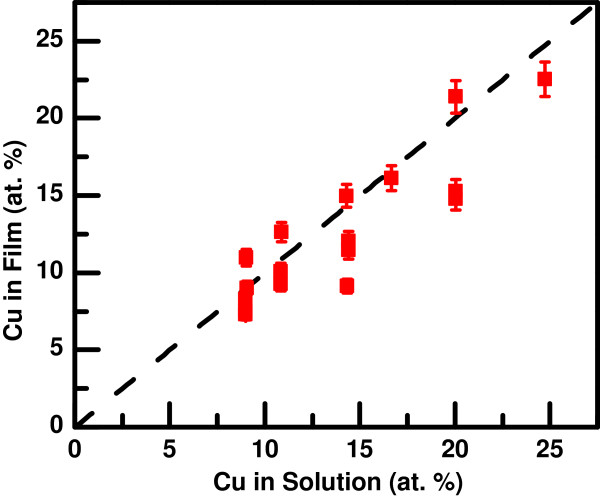
**Copper composition in electrodeposited NiCu thin films.** Copper composition in the electrodeposited films as determined by EDS as a function of the copper composition in the deposition solution. Each point represents a single sample, and the error bars are the typical EDS uncertainty. The dashed line indicates equal composition in the solution and in the film.

To characterize the results of both the deposition and dealloying steps, the composition of the as-deposited and dealloyed films was measured using EDS. Figure 1 shows the Cu concentration (in atomic %) of the deposited NiCu films as a function of the corresponding Cu concentration in the deposition solution. Each point in the graph represents a single sample, and the error bars are the typical uncertainty for the EDS measurements. The dashed line indicates the case that the film composition is equal to the solution composition. At the deposition potential of -1,200 mV, the deposition rates for both Ni and Cu are essentially diffusion-controlled, so the composition of the films track the composition of the solutions to a large extent. However, there is some variation in the results from sample to sample, reflecting a degree of variability in the experimental setup.

**Figure 2:**
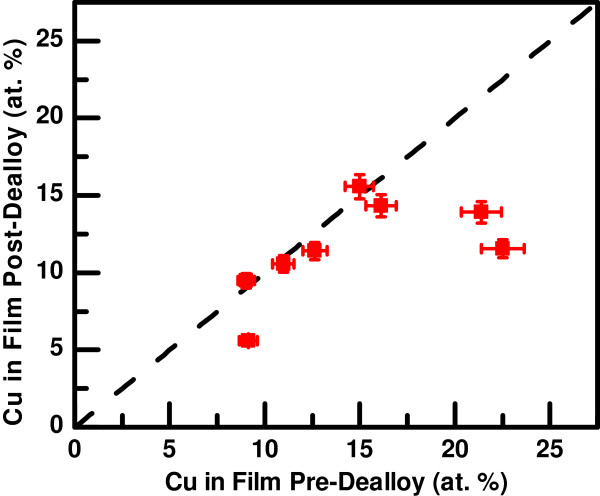
**Copper composition in dealloyed NiCu thin films.** Copper composition in the dealloyed films as a function of the composition in the as-deposited film. Each point represents a single sample, and the error bars are the typical EDS uncertainty. The dashed line indicates removal of both components at equal rates.

The effect of the dealloying procedure on the Cu content of the samples is shown in Figure 2, where the Cu composition after dealloying is compared to the composition in the as-deposited films. Again, each point represents a single sample, and the error bars indicate the typical uncertainty for the EDS measurements. The dashed line indicates no net change in the Cu composition, that is, removal of both species at identical rates. Over the range of Cu concentrations studied, one of two outcomes was achieved. Either both species were removed at the same rate, so that statistically the post-dealloy Cu composition did not change, or Cu was selectively removed, leading to a decrease in the Cu composition. For higher initial Cu concentrations, copper was selectively removed. However, for the LSV dealloying procedure used, there is evidence of a lower limit to the Cu removal, resulting in samples with about 12% Cu.

**Figure 3:**
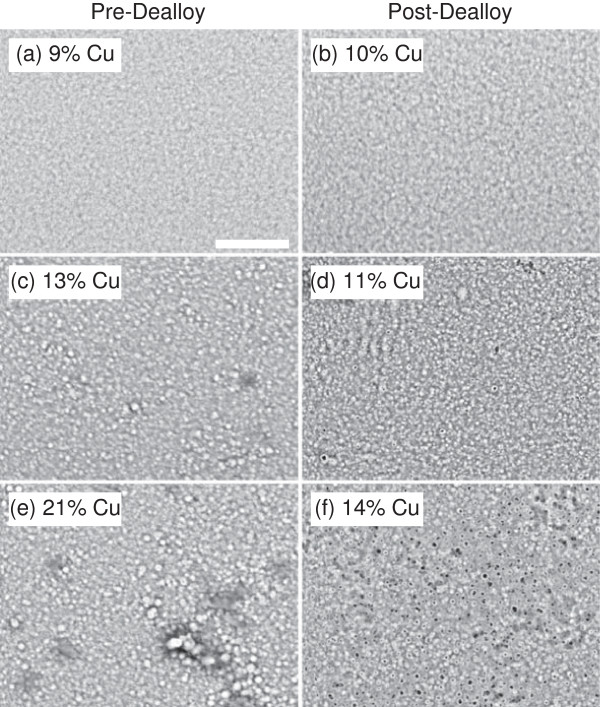
**SEM images of NiCu films before (a, c, e) and after (b, d, f) the dealloying procedure.** The initial copper content in the films are (a) 9.0±0.5%, (c) 12.6±0.6%, and (e) 21.4±1.1%. The copper content in the dealloyed films are (b) 9.5±0.5%, (d) 11.4±0.6%, and (f) 13.9±0.7%. The scale bar is 5 *μ*m for all the images.

**Figure 4:**
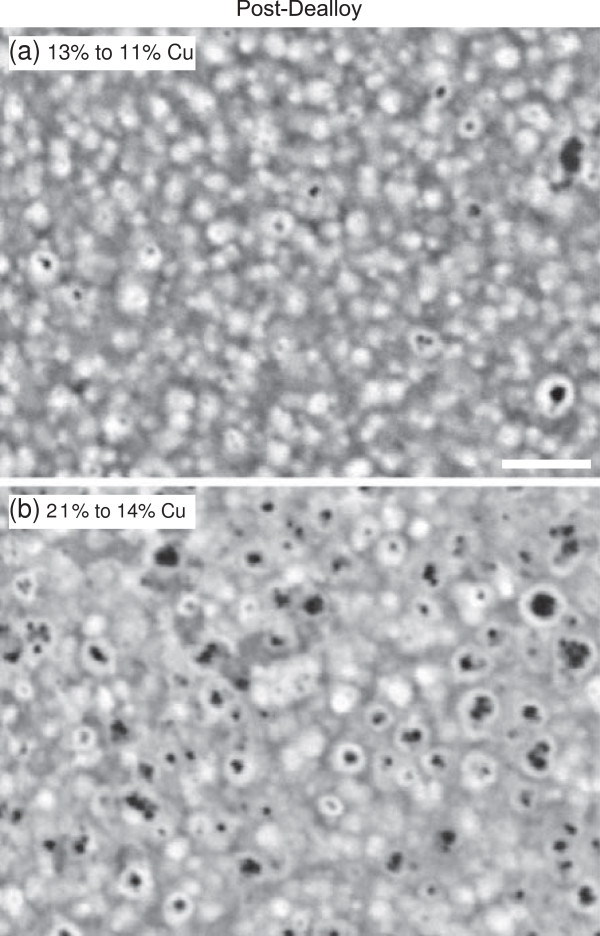
**Higher resolution SEM images of the dealloyed NiCu films in (a) Figure **3**d and (b) Figure **3**f.** The scale bar is 1 *μ*m for both images.

The structure of the as-deposited and dealloyed NiCu samples was characterized using SEM. Example SEM images of the NiCu films are shown in Figure 3 both before (a, c, e) and after (b, d, f) the dealloying procedure. As the initial copper content in the film increases (from a to c to e), the grain size and roughness of the as-deposited film increases slightly. For the case of 9% Cu in the as-deposited film (a), the dealloying procedure left the Cu concentration essentially unchanged, and structure of the film is also largely the same (b). For the films with 13% and 21% Cu (c and e), the dealloying procedure decreased the copper content in the film and resulted in surface pits where copper was removed (d and f). The pits formed in the sample with the smaller initial Cu concentration (d) are smaller than those formed in the sample with the larger initial Cu concentration (f). This can be seen more clearly in the higher resolution SEM images of the post-dealloyed films in Figure 4.

**Figure 5:**
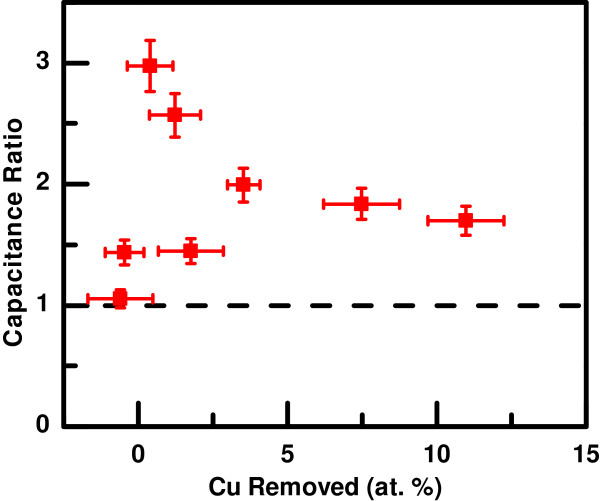
**Ratio of measured capacitance after to before the dealloying step.** The capacitance ratio as a function of the copper composition (at.%) removed in the dealloying step. Negative Cu removed indicates that Ni was selectively removed in the dealloying step rather than Cu. The dashed line indicates identical measured capacitances before and after dealloying.

To compare the resulting electrochemically accessible surface areas of the samples, the electrochemical double-layer capacitance was measured for each sample both before and after the dealloying step. In the simplest model, this capacitance is proportional to the surface area of the sample accessible via electrochemistry and thus provides a semi-quantitative measure of that area. Figure 5 shows the ratio of the measured capacitance after the dealloying step to before the dealloying step as a function of the amount of copper selectively removed. In the figure, negative Cu removed indicates that Ni was selectively removed in the dealloying step; for these samples, when the uncertainties are taken into account, the Cu removed amounts are statistically equivalent to zero. The dashed line indicates identical measured capacitances before and after dealloying.

For all the samples studied, the capacitance either stayed statistically the same or increased, suggesting that the dealloying procedure either did not change the effective surface area of the sample or caused it to increase. For the samples with between 3% and 15% Cu removed, the capacitance ratio decreases as the amount of copper removed increases. This observation is consistent with the SEM images in Figures 3 and 4. The samples with larger initial copper content tended to have rougher initial topography, such as that in Figure 3e, and thus had higher initial capacitance measurements. In addition, those samples tended to have larger pits seen in the post-dealloy topography, such as in Figure 3f, which increased the measured capacitance only modestly. For the samples with smaller amounts of copper removed, there is more variation in the resulting capacitance ratio. The largest increases in capacitance occurred for samples with a moderate initial copper content combined with a small amount of copper removal, resulting in numerous small pits in the post-dealloy topography. The largest capacitance ratio observed for these samples implies a factor of 3 increase in surface area after dealloying.

### Hydrogen evolution reaction measurements

**Figure 6:**
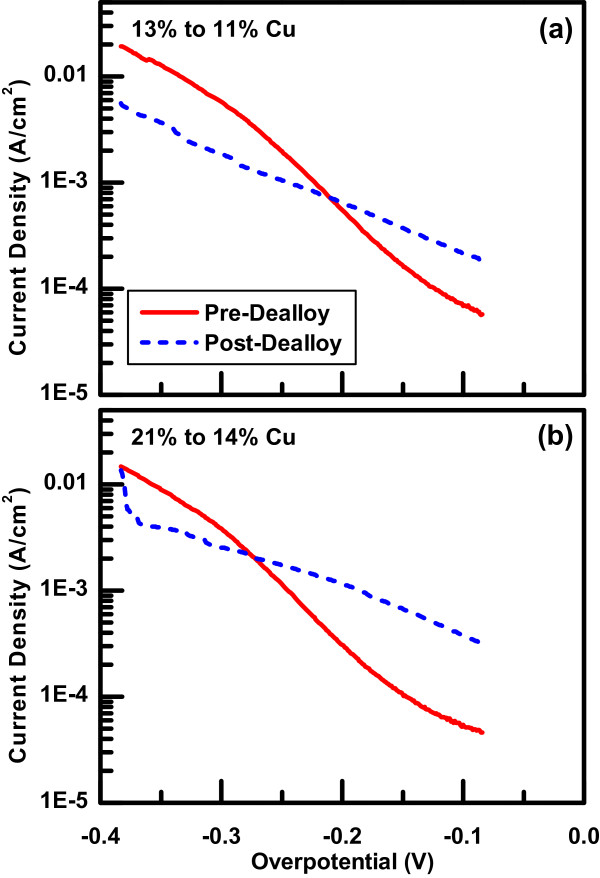
**HER measurements of two samples both before and after the dealloying process.** Current densities were calculated with respect to the geometric area of the sample. The initial copper content in the films are **(a)** 12.6±0.6% and **(b)** 21.4±1.1%. The copper content in the dealloyed films are (a) 11.4±0.6% and (b) 13.9±0.7%.

To characterize the catalytic behavior of the samples, HER measurements were made both before and after dealloying. Example Tafel plots of the data are shown in Figure 6. In general for these samples, the HER current density is larger after dealloying for low overpotentials, but smaller after dealloying for larger overpotentials. That is, the dealloyed samples are more reactive at lower overpotentials but less reactive at higher overpotentials for HER measurements. In addition, the data show a range of Tafel slopes for the overpotential range measured. This effect is more significant for the as-deposited samples.

**Figure 7:**
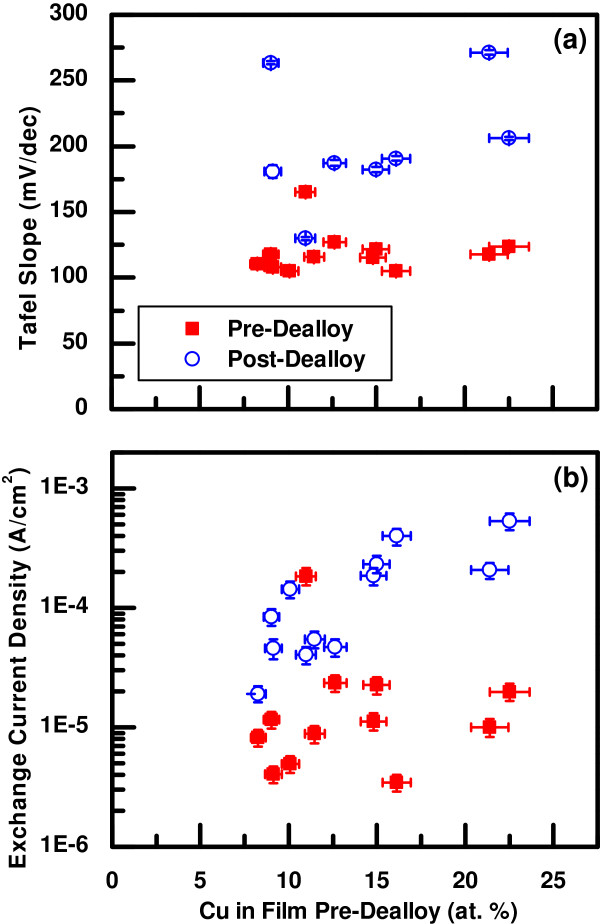
**Tafel slope and current density extracted from HER measurements.(a)** Tafel slope and **(b)** exchange current density from HER measurements of the as-deposited and dealloyed NiCu thin films as a function of Cu content in the film before dealloying.

For each set of measurements, the high overpotential data (between -350 and -200 mV) were fit to the Tafel equation, *J* = *J*_0_*e*^−*B* *η*^, where *J* is the current density and *η* is the overpotential. The Tafel slope, $b=\frac{\ln(10)}{B}$, and exchange current density, *J*_0_, were determined from the fit parameters. The results are shown in Figure 7 as a function of the Cu composition initially in the sample. Consistent with the data in Figure 6, the samples tend to have both higher Tafel slope and higher exchange current density after dealloying compared to their as-deposited counterparts. This combination causes the crossing of the HER curves in Figure 6, where the dealloyed samples are more reactive at lower overpotentials and less reactive at higher overpotentials.

For the as-deposited samples, the Tafel slopes tend to be around 100 to 125 mV/dec. In contrast, the Tafel slopes for the dealloyed samples are generally higher, most above 175 mV/dec. One possible reason for these larger Tafel slopes is a decrease in effective area available for reaction at higher overpotentials due to larger gas evolution rates. This effect may be increased by the more porous nature of the dealloyed samples, allowing gas bubbles to be trapped more easily. To confirm this hypothesis, additional measurements of the effective surface areas at different applied potentials during HER conditions are needed.

The exchange current densities for the as-deposited samples were generally lower than those for the dealloyed samples. The increase in exchange current density for the samples after dealloying is more pronounced (over an order of magnitude) for the samples with larger initial Cu content. This increase cannot be explained purely by an increase in effective surface area. The measured capacitances generally increased by a factor of 2 to 3 after dealloying (Figure 5), so the additional increase in reactivity must be due to structural and compositional changes in the thin films.

## Conclusions

Electrodeposition and electrochemical dealloying of NiCu thin films were used to fabricate porous samples. The hydrogen evolution reactivity of electrodeposited NiCu samples was measured before and after some of the Cu was selectively removed. The dealloyed samples are generally more reactive at lower overpotentials, but less reactive at higher overpotentials. The increase in reactivity for the dealloyed samples, as measured by the exchange current density, cannot be explained only by an increase in effective surface area. Thus, some of the reactivity increase must be due to the changes in composition and structure of the samples from the dealloying procedure. The decrease in reactivity at higher overpotentials is hypothesized to be the result of trapped hydrogen bubbles decreasing the effective surface area of the samples. Further experiments are ongoing in our laboratory to investigate the effective surface area of as-deposited and dealloyed samples as a function of potential. The dealloying procedure used here is a promising method for the fabrication of effective catalysts for HER, particularly for use at low overpotentials.

## Competing interests

The authors declare that they have no competing interests.

## Authors’ contributions

KRK and EFN carried out the experiments and contributed to the data analysis. JRH coordinated the study and helped analyze the data. All authors helped draft the manuscript and approved its final form.
